# Long term studying of uranium and radium-226 activity in drinking water in some regions of Ukraine and assessment of corresponding hypotetical irradiation doses

**DOI:** 10.1038/s41598-024-53079-z

**Published:** 2024-01-30

**Authors:** Mykhailo Buzynnyi, Liubov Mykhailova

**Affiliations:** State Institution O.M. Marzieiev Institute of Public Health of National Academy of Medical Sciences of Ukraine, 50, Hetman Pavlo Polubotko Str. (Popudrenko), Kyiv, Ukraine

**Keywords:** Freshwater ecology, Ecology, Environmental sciences, Medical research

## Abstract

The article summarizes the activity concentrations data of ^226^Ra and the sum of uranium isotopes (∑U) in samples of drinking underground water for different regions of Ukraine studied during 1998–2023 in the radiation monitoring laboratory of the State Institution "O.M. Marzieiev Institute of Public Health National Academy of Medical Sciences of Ukraine. Arithmetic mean and standard deviations, minimum and maximum values for ^226^Ra and ∑U activity concentrations are presented for the entire 1240 sample set and for each region separately. Collected data show that the established state permissible level for drinking water of 1.0 Bq/l is exceeded for ^226^Ra in 1.1% of the studied samples, and for ∑U—in 3.9% correspondingly. The detected high levels of ^226^Ra and ∑U activity concentrations correspond to certain regions belonging to the Ukrainian crystalline shield territory. A comparison of the current data with the data of previous studies held during of 1989–1991 indicates a significant difference: for the previous studies the average and standard deviations are much higher. We attribute this to the fact that the centralized sampling of previous studies was random, and it was related exclusively to communal water supply systems. At the same time, the current sample set covers a much larger number of regions, different water consumers; the data set includes the results of repeated studies for a large number of sources, in particular, sources with purified water. Hypothetical exposure doses caused by consumption of ^226^Ra and ∑U in water for the current sample set were estimated for different age groups for each sample studied, as is, without taking into account the pattern of water consumption. The corresponding dose exceeds the WHO recommended value of 0.1 mSv per year for children under the age of one year for 220 cases (17.7%). This dose limit excess for other age groups corresponds—for children: aged 12–17 years—13.1%, aged 1–2 years—7.4%, 7–12 years old—5.6%, 2–7 years old—3.9% and for adults—4.1%.

## Introduction

Protection of underground water is commonly much higher than for surface water with regard to chemical and microbiological pollutants, which sets the priority of its use as drinking water source^1^. At the same time, underground water contains natural radionuclides of the U and Th series. The radioactivity concentration in water is formed by the radioactive composition of the rocks, the level of fissuring of the rocks, the chemical properties of the water and the contact time of the water with the rocks^2^. Author shoved scatted plot of uranium versus ^226^Ra in random Australian ground water illustrating the lack of correlation between parent and decay product. The introduction of natural radionuclides into water occurs due to diffusion of gas (^222^Rn) or leaching (U, Ra) from the surface of rocks. The chemical composition of water defines the intensity and priority of certain radionuclides leaching.

High levels of natural radionuclides in drinking water are observed primarily for artesian wells with a depth of several tens to hundreds of meters, although in some places favorable conditions for the entry of natural radionuclides into water present even for aquifers with a depth of only a few meters or even for surface streams^3^. In addition, high levels of natural radionuclides in surface water can form discharges from mining and processing industrial enterprises, mines.

Approaches to the national monitoring programs development for radioactivity of potable water and the selection of mandatory components have been formed historically (NRBU-97, EPA, Chen et al. 2018, Vesterbacka et al. 2005)^4–7^. Recently worldwide, in particular in Ukraine^8^, measurement of total alpha and total beta activity has been introduced as an essential element of mass screening of water radioactivity, as it may simplify research, shorten time and save money. At the same time, such studies reveal a special role—a "false alarm" of high alpha activity caused by ^224^Ra, which is not accompanied by ^228^Ra^9^ and the necessity to determine ^40^ K to exclude its contribution when the total beta activity exceed permissible level.

Drinking water is an important route of natural radionuclides intake into the human body, forming natural exposure, which is difficult to avoid^5^. The most significant contributors of the water radioactivity are ^238^U and ^234^U, ^226^Ra, ^228^Ra, ^222^Rn, ^210^Pb and ^210^Po. In most cases, exposure due to the water radioactivity is a combination where main contributors include ^222^Rn. Natural radionuclides of the uranium series are, as believed, making the main contribution to exposure due to water consumption. ^222^Rn forms a significant inhalation component of exposure due to its entry into the air of residential premises. Harm to human health from water consumption is caused its radioactivity, in particular, uranium and radium isotopes. At the same time chemical toxicity of uranium, primarily ^238^U, can wrongly be estimated like in our case, when known is only the ∑U activity concentration without knowing ^234^U/^238^U activities ratio^10^.

Assessments of human exposure to radionuclides contained in drinking water have long be receiving considerable attention worldwide (EPA, Council Directive, 1998, UNSCEAR, 2000, 2013/51/EURATOM, 2013, WHO, 2018)^5,11–14^. Relevant studies cover certain geographical areas. They take into account natural landscape features and geology anomalies and cover role of industrial impact on water sources contamination. Study were held to evaluate the radioactivity of water as a commercial raw material^15–20^ or as a market end-product sold in a retail network^21–24^. Practically all modern water radioactivity studies include estimation of the irradiation dose to the population caused by this water consumption, focusing in particular on the use of age-dependent dose factors (Council Directive 96/29/Euratom)^25^.

### Regulations of water radioactivity in Ukraine

National radiation safety standard was created in 1997^4^ and it covers natural radioactivity of water: ^222^Rn—100 Bq/l, ^226^Ra—1.0 Bq/l, ∑U—1.0 Bq/l. Corresponding water sources to be studied supposed to be wells or groups of wells operating together. Later in 2010, sanitary regulations corresponding to the radioactivity of water were updated as Ref.^8^, requiring measurement of total alpha- and total beta- activity (permissible levels 0.1 and 1.0 Bq/l, respectively), and only if there is an excess, to do measurement of uranium and radium. ^222^Rn test requires anyway for every water sample tested in lab. The preparing to release of a number of Ukrainian agricultural and food products to the EU market caused the application of the requirements of the Council Directive 98/83/EC^11^, and more recently the Council Directive 2013/51/EURATOM^13^ to the relevant Ukrainian agricultural and processing producers. Thus, the need arose to conduct local relevant studies of the water radioactivity. So in all above requirement options^4,8,11,13^, we had worked with water radioactivity and accumulated the data of ^226^Ra and ∑U isotopes activity in drinking water made to order and finally consider them now.

### Studies of natural water radioactivity in Ukraine

In Ukraine, the research of natural radionuclides in water for the purpose of geological exploration of minerals was carried out since the 40^ s^ of the last century. Later some geological features of ^222^Rn and ^226^Ra formation and distribution in the ground water were studied and described by Ref.^4^.

**Figure 1:**
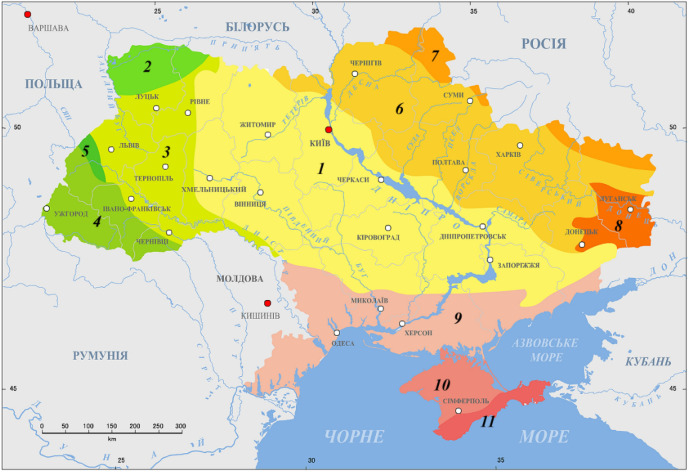
Tectonic structure of Ukraine: 1. Ukrainian Shield; 2. Kovel salient; 3. Volyn-Podilla plate; 4. Carpathian fold and thrust belt; 5. West-European platform; 6. Dnieper-Donets rift; 7. Voronezh massif; 8. Donets fold belt; 9. Black Sea depression; 10. Scythian plate; 11. Crimean fold and thrust belt^27^.

Our previous studies of ^222^Rn in drinking water show us significant dose forming role, especially when water supplied into residential premises, which made it possible for us understand and determine the water-to-air transfer coefficient^26^. The results of comprehensive hygienic studies of natural radionuclides radioactivity in a statistically weighted sample set of drinking water samples (> 1400 samples) in Ukraine conducted in the early 1990s were presented by Ref.^1^. These studies covered five regions within the Ukrainian Crystalline Shield^27^, see Fig. 1, and three regions outside of it, for comparison. The obtained data proved the extreme heterogeneity of the natural radionuclides concentration in underground waters of different regions of Ukraine. The maximum activity of ^222^Rn for the territories belonging to the Ukrainian shield reached 2660 Bq/l compared to 195 Bq/l outside the shield. For ^226^Ra, the maximum values on the shield’s territory were 5.3 Bq/l compared to 0.24 Bq/l outside the shield, and for uranium, the maximum values reached 21.3 Bq/l on shield, compared to 0.77 Bq/l outside the shield (Zelenskyi et al., 1993)^2^. So, authors showed that the activity of natural radionuclides in water is significantly higher within the Ukrainian Crystalline Shield than outside of it.

These data^1^ actualized the existing problem, encouraged us to consider natural radioactivity of artesian water as an important factor in shaping the exposure of the population of Ukraine and became the basis for the development of national radiation standards for drinking water^4,8^. The data and the given estimates of expected exposure doses attracted attention even during their presentation at the conference^1^, and they became the background for the discussion of the following results presented later^28–31^.

Among sample set of^1^, about 100 was investigated for Th and U using inductively coupled mass spectrometry^10^. Corresponding data confirmed the data^1^ on the uranium isotopes concentration and, in addition, drew attention to the high ^234^U/^238^U activity ratio, which sometimes reached here level of 10–20 and higher.

Recently, we summarized the radon concentration data in water studied for the period 1998–2022^32^, where estimates were made for the entire sample set and for its parts corresponding to different regions of Ukraine and, in addition, obtained for separate consecutive time intervals.

In addition, we had recently received interesting signal results^33^ in the course of water research accompanying the installation of individual water purification systems in private homes in the city of Zhytomyr and suburb, an area that belongs to the Ukrainian Crystalline Shield^27^. According to the results of the study of 20 samples taken with the consent of the owners, in 17 cases (85%) the level of ^222^Rn exceeds 100 Bq/l, when uranium only in 2 cases (10%), and ^226^Ra only in 1 case (5%) exceed the permissible level of 1.0 Bq/l^4,8^.

## Methods

### Water sample set

Here we have been analyzing the data of natural radionuclide activity concentration in drinking water from the territory of several regions of Ukraine, which have been studied out since 1998 at the request of the owners regarding water compliance with the requirements of the standards^4,8^, as well as lately European legislation^11,13^, around 150 samples.

Part of the samples of the general sample set was studied according to indicators of total alpha and beta activity^31,34^, however, in the vast majority of samples we studied ^226^Ra and ∑U. So, in this work, we consider the activity concentration of ^226^Ra and the ∑U in 1240 water samples.

A subset of 62 water samples data was also presented here, which were recently studied to support the implementation of individual water purification systems in the Zhytomyr region as a development of the previously presented research^33^.

### Methods of determining activity concentration

To determine the activity concentration of ^222^Rn, ^226^Ra and the sum of uranium isotopes, we had used liquid scintillation spectrometer Quantulus 1220™ and extractive sample preparation methods. Our scintillation "cocktail" was based on PPO (4 g/l) and POPOP (0.1 g/l) dissolved in toluene or o-xylene.

For ^222^Rn measurement we initially place 10 ml of our scintillation "cocktail" in 20 ml glass vial, and then we placed there 10 ml of the water sample. We cover and close vial tightly and shake it for a minute. Sample is ready for measurement after 10 min of equilibration of two-phase sample, we always used the part of the alpha spectrum corresponding to ^222^Rn + ^218^Po. In 2019, we participated in comparative tests for radon measurements in water (Jobbagy et al. 2019, Lab code #27)^35^. We obtained acceptable results, both for water miscible and immiscible scintillator used especially considering that the water sample arrived at the laboratory with a delay of more than 7 days.

When calculating the ^222^Rn activity of the samples (see Formula (1)), we took into account the actual water sample volume (V = 10 ml) and the ^222^Rn decay for the time interval since sampling to the moment of measurement.

1 $${A}_{Rn}=\frac{(CPM-BG)\cdot {e}^{\frac{\mathrm{0,693}\cdot \Delta T}{\mathrm{3,82}}}}{E\cdot V\cdot 60} (Bq/1);$$

A_Rn_—^222^Rn activity concentration; CPM—Sample count rate (count per minute); BG—Background sample count rate (count per minute); ΔT—Sample delay time interval, since sampling to counting; 0.693 = log(2); E—Counting efficiency, for counting window corresponding to ^222^Rn + ^218^Po (~ 200%); V—Corresponding water sample volume, L.

To determine ^226^Ra activity concentration, we used 100–200 ml of water, which we evaporate to dryness in heat resistant glass vessel. Evaporation was performed with heat above middle of electric plate or using of sand bath. The residue we dissolve in 4 ml (2 ml) of a 1 M HNO_3_ solution, and then put it into a Teflon vial (20 ml or 7 ml). Finally we rinsed the glass vessel again with 4 ml (2 ml) of distilled water, which was also introduced into the same Teflon vial where then we placed at the end 12 ml (4 ml) of mentioned above scintillation "cocktail", which is immiscible with water. The Teflon vial was closed with a tight cap, and the measurement of ^222^Rn accumulated in the solvent with ^226^Ra was carried out in two phase sample no earlier than after 7–10 days^29^.

When calculating the ^226^Ra activity of the samples (see Formula (2)), we took into account the actual volume of the water sample used and the ^222^Rn/^226^Ra equilibrium coefficient achieved at the time of measurement, which, for the above mentioned time interval, was 72–84%.

2 $${A}_{Ra}=\frac{(CPM-BG)\cdot {(1-e}^{-\frac{\mathrm{0,693}\cdot \Delta T}{\mathrm{3,82}}})}{E\cdot V\cdot 60}(Bq/1);$$

A_Ra_—^226^Ra activity concentration; CPM—Sample count rate (count per minute); BG—Background sample count rate (count per minute); ΔT—Sample delay time before counting; 0.693 = log(2); E—Counting efficiency which depends on width of counting window corresponding to ^222^Rn + ^218^Po (~ 200%) or ^222^Rn + ^218^Po + ^214^Po (~ 300%); V—Corresponding water sample volume, L.

Periodically, we check the quality of sample material washing ^226^Ra from the walls of the glass beaker in the process of sample preparation after evaporation of water to dryness. For this, we use two identical washings of the sample from the beaker (see above), performed one after the other. The recently performed test showed that when the sample is concentrated from 100 ml of mineralized water, which has a dry residue of 1.12 g per liter, 3.3% ± 0.9% of the activity of the sample remains in the second consecutive sample (we had used 3 repeats). Such a high amount of dry residue is more characteristic for mineral water, but sometimes it is also occur in the studied drinking water. We used a similar control procedure while the participation in laboratory intercomparison tests on measurements of total alpha- and total beta- activity of two water samples (dry residue of 0.37 and 0.96 g/l) organized by the JRC EC, our Lab code #17537^36^.

It should be added that for certainty in the results of measurements of ^226^Ra in water, it is very important to test Teflon vials for their tightness. For this we determine the individual loss of the organic phase mass of the sample during the time before its measurement (7–10 days), or in a separate check of the set of vials, without water phase, when the entire volume is occupied by the organic phase, and the waiting period is 3–5 weeks.

To study the activity concentration of ∑U, we used the method described earlier^29^. It includes the water sample pre-concentration from 1 L, for which to the water sample in heat-resistant beaker we added 5 ml of FeCl_3_ solution (~ 10 mg/ml on iron) and 5 ml of HNO_3_ and brought it to a boil. Next, we added 10 ml of ammonia solution (25%) to the beaker for co-precipitation of uranium with iron hydroxide. We cooled the water at air, and after about 1 h, the precipitate was first vacuum filtered on a paper filter, and then it was dissolved in 40 ml of 6 M HNO_3_ solution.

Next, uranium was extracted (3 min) in 10 ml of a 20% solution of tributyl phosphate in toluene or o-xylene. The obtained organic phase was washed twice (3 min each) from acid residues with 20 ml of 6 M NH_4_OH solution, and finally bubbled with argon (3 min). For measurements, the obtained sample was placed in a 20 ml plastic vial, where 10 ml of the scintillation "cocktail" mentioned above was added. The average chemical yield of uranium was 65 ± 3%. For each sample, we monitored losses of extracting solution by weighing the organic phase after the preparing was complete. We calculated the activity of the samples according to Formula (3).

3 $${A}_{U}=\frac{(CPM-BG)\cdot \frac{{m}_{{e}_{i}}}{{m}_{e}}}{E\cdot V\cdot {C}_{y}\cdot 60}(Bq/1);$$

A_U_—activity concentration for ∑U; CPM—sample count rate (count per minute); BG—background count rate (count per minute); C_y_—base extraction empirical parameter (65%) defined by 20% TBP in toluene extraction solution and water (HNO_3_) to organic phases ratio 40: 10; m_ei_—initial mass of extraction solution added; m_e_—final mass of extraction solution extracted; E—counting efficiency (~ 100%); V—Water sample volume, L.

The extraction method we use for uranium measurements is implemented under the condition of highly selective co-precipitation of uranium with iron hydroxide at pH 9, which gives a moderate extraction efficiency of about 65%. For quality control and determining the efficiency of the method, a calibrated solution of ^232^U (1.0 Bq/ml) and/or a laboratory solution of uranyl nitrate are used. Freshly extracted sample, ^232^U gives a single high-resolution alpha peak (without daughter products), which we use to determine the efficiency of extraction, and later the spectrum changes with the appearance and accumulation of daughter products. Under using uranyl nitrate, we have the opportunity to determine its activity in a water solution sample, when the extracted sample shows, in particular, how selectively uranium is extracted in relation to ^234^Th and ^234^ Pa (both beta emitters) and we also use it to determine the efficiency of extraction. This is the depleted uranium calibration sample we use, and it has a ratio of ^234^U/^238^U < 1.0, which is different from what we observe for groundwater samples.

When using the above specified sample preparation scenarios and taking into account a measurement time of 180 min, the minimum detected activity (MDA) is equal to 0.004 Bq/l for uranium and 0.005 Bq/l for ^226^Ra, according to formulae Formula (4)^37^. MDA for ^222^Rn measurement calculated for 20 min was 0.5 Bq/l.

4 $$MDA=\frac{2\cdot \sqrt{BG\cdot T}}{M\cdot T\cdot Error\cdot 60\cdot Eff}$$

MDA—minimum detected activity; BG—background count rate, CPM (0.15 for ∑U and 0.01 for ^226^Ra); T—counting time, min; M—sample volume, L; Error—uncertainty, percent (30%); 60—time units conversion factor, sec/min; Eff—counting efficiency.

Usually, while Quantulus 1220™ measurements, we use the parameter of Pulse Shape Analyzer (PSA) equal 60 for ^222^Rn and ^226^Ra measurements in a two-phase sample (Teflon vial) and PSA = 30 for measurements of uranium (plastic vial), but in some cases when samples were quenched, measurements had to be repeated under other PSA conditions. We should note that in recent years it was mainly used o-xylene as a solvent.

## Methodology of dose estimation

Annual dose Dx for age group x is calculated from the expression, see Formula (5):

5 $${D}_{x}=\sum_{i}{C}_{x}\cdot {A}_{i}\cdot {(DCF)}_{x}$$

where: *C*_*x*_ is the annual water consumption for an age group *x* (L/a), *A*_*i*_ is the activity concentration of nuclide *i* (Bq/L), *(DCF)*_*ix*_ is the dose conversion factor (dose coefficient) for nuclide *i* and age group *x* (mSv/Bq).

The lifetime average annual dose associated with a water resource is calculated from the expression, see Formula (6):

6 $$D=\sum_{i}{A}_{i}\cdot {F}_{i}$$

where: *D* is the lifetime average annual dose (mSv/a), *A*_*i*_ is the activity concentration of nuclide *i* (Bq/L), *F*_*i*_ is a proportionality constant for nuclide *i* with units of (mSv/a) per (Bq/L).

The proportionality constant *F*_*i*_ for nuclide *i* was determined from the following relationship, see Formula (7):

7 $${F}_{i}=\sum_{i}{C}_{x} {(DCF)}_{ix}{W}_{x}$$

where: *C*_*x*_ is the annual water consumption for an age group *x* (L/a), *W*_*x*_ is a weighting factor for age group *x*.

The weighting factor for each age group was determined by dividing the number of years in the age group by the average life expectancy, taken to be 70 years. For example, the weighting factor for the 7–12 years age group was, see Formula (8):

8 $${W}_{7-12}=\frac{12-7}{70}=0.0714$$

## The results and discussion

**Figure 2:**
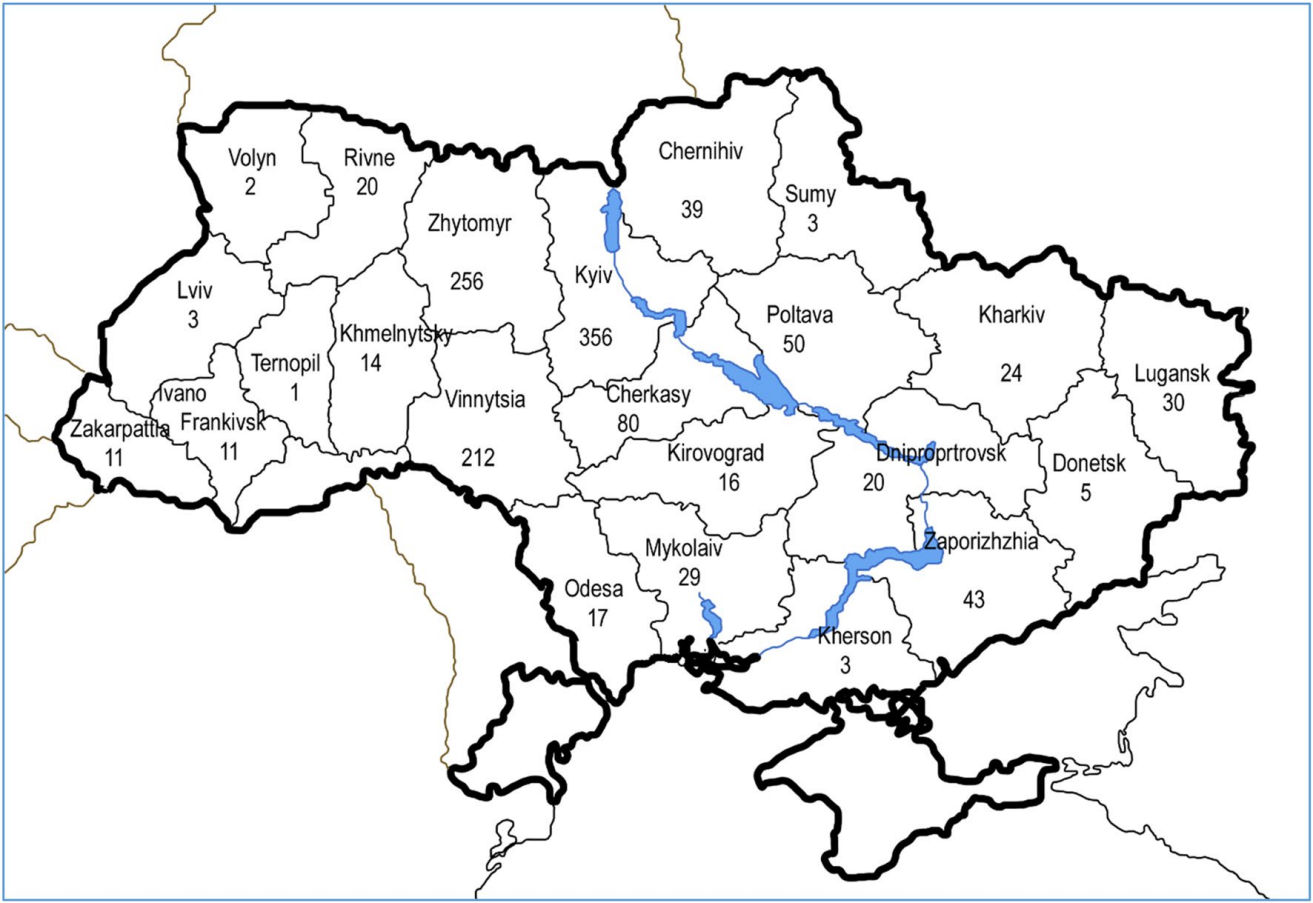
Water sample set by regions on map of Ukraine, where we use map^38^ as a blank.

Figure 2 shows how water sample set covers territory of Ukraine. It is clearly seeing that most representative are three regions, closest to laboratory location: Kyiv, Zhytomyr and Vinnytsia, which are located on Ukrainian Shield^27^.

### Radioactivity of water samples

In Table 1 are given generalized data (number, sampling period, average, standard deviation, median, interquartile range, minimum and maximum) for ^226^Ra and ∑U activity concentration in total set and by region, taking into account the corresponding sample number for each region (descending order). Maximum values exceeding the permissible level of 1.0 Bq/l according to national radiation safety standard (NRBU-97 and SanPiN-2010)^4,8^ are highlighted in bold.

**Table 1:** Generalized data for ^226^Ra and ∑U activity concentration (Bq/l) in water samples.

Region (oblast)	N	Period	^226^Ra	Sd	Median	IQR	Min	Max	∑U	Sd	Median	IQR	Min	Max
Kyiv	356	1999–2023	0.04	0.096	0.014	0.024	0.002	0.88	0.13	0.46	0.014	0.052	0.001	**6.3**
Vinnytsia	251	1998–2023	0.025	0.034	0.015	0.019	0.001	0.37	0.17	0.23	0.10	0.17	0.006	**1.6**
Zhytomyr	212	1999–2023	0.26	0.60	0.08	0.19	0.002	**3.6**	0.27	0.67	0.05	0.17	0.002	**8.1**
among them	62	2020–2023	0.23	0.43	0.047	0.22	0.005	**3.1**	0.22	0.47	0.12	0.15	0.003	**2.8**
Cherkasy	80	2006–2023	0.102	0.14	0.048	0.12	0.005	0.86	0.52	0.58	0.21	0.91	0.01	**2.3**
Poltava	50	2004–2023	0.025	0.023	0.013	0.036	0.002	0.08	0.021	0.025	0.012	0.01	0.004	0.13
Zaporizhzhia	43	2005–2023	0.023	0.032	0.012	0.015	0.002	0.2	0.33	1.20	0.20	0.044	0.001	**6.2**
Chernihiv	39	2004–2019	0.014	0.010	0.01	0.012	0.004	0.05	0.027	0.046	0.01	0.014	0.002	0.21
Lugansk	30	2001–2021	0.020	0.037	0.009	0.007	0.004	0.2	0.089	0.11	0.05	0.081	0.002	0.42
Mykolaiv	29	2001–2023	0.044	0.11	0.019	0.024	0.005	0.58	0.087	0.17	0.014	0.037	0.003	0.6
Kharkiv	24	2008–2023	0.016	0.022	0.009	0.013	0.002	0.11	0.098	0.19	0.016	0.037	0.004	0.63
Rivne	20	2005–2023	0.024	0.019	0.019	0.021	0.005	0.08	0.050	0.13	0.013	0.02	0.008	0.61
Dnipropetrovsk	20	2008–2023	0.040	0.11	0.011	0.015	0.004	0.49	0.13	0.28	0.03	0.054	0.008	**1.24**
Odesa	17	2003–2023	0.017	0.017	0.01	0.016	0.004	0.06	0.027	0.031	0.02	0.02	0.006	0.14
Kirovograd	16	2009–2023	0.18	0.17	0.012	0.22	0.005	0.56	0.20	0.14	0.22	0.27	0.04	0.39
Khmelnytskyi	14	2009–2016	0.038	0.016	0.042	0.023	0.006	0.06	0.011	0.012	0.007	0.005	0.004	0.05
Ivano Frankivsk	11	2009–2023	0.009	0.006	0.006	0.007	0.002	0.02	0.020	0.013	0.017	0.016	0.005	0.05
Zakarpattia	11	2003–2020	0.25	0.31	0.008	0.57	0.005	0.72	0.085	0.15	0.011	0.096	0.002	0.52
Donetsk	5	2004–2023	0.044	0.043	0.02	0.073	0.005	0.1	1.17	2.40	0.14	1.50	0.017	**5.5**
Sumy	3	2009–2022	0.008	0.003	0.007	0.005	0.005	0.01	0.19	0.31	0.01	0.41	0.005	0.55
Kherson	3	2006–2020	0.056	0.051	0.05	0.077	0.008	0.11	0.59	0.91	0.092	1.19	0.05	**1.6**
L’viv	3	2017–2020	0.011	0.008	0.006	0.011	0.006	0.02	0.071	0.04	0.074	0.06	0.03	0.11
Volyn	2	2018–2021	0.013	0.011	0.013	0.015	0.005	0.02	0.033	0.024	0.033	0.034	0.016	0.05
Ternopil	1	2005	0.04						0.099					
Total	1240	1998–2023	0.078	0.274	0.018	**0.038**	0.001	**3.64**	0.18	0.51	0.03	**0.13**	0.001	**8.10**

Significant values are in bold.

### Support of installation of water purification systems in Zhytomyr city and suburb

**Figure 3:**
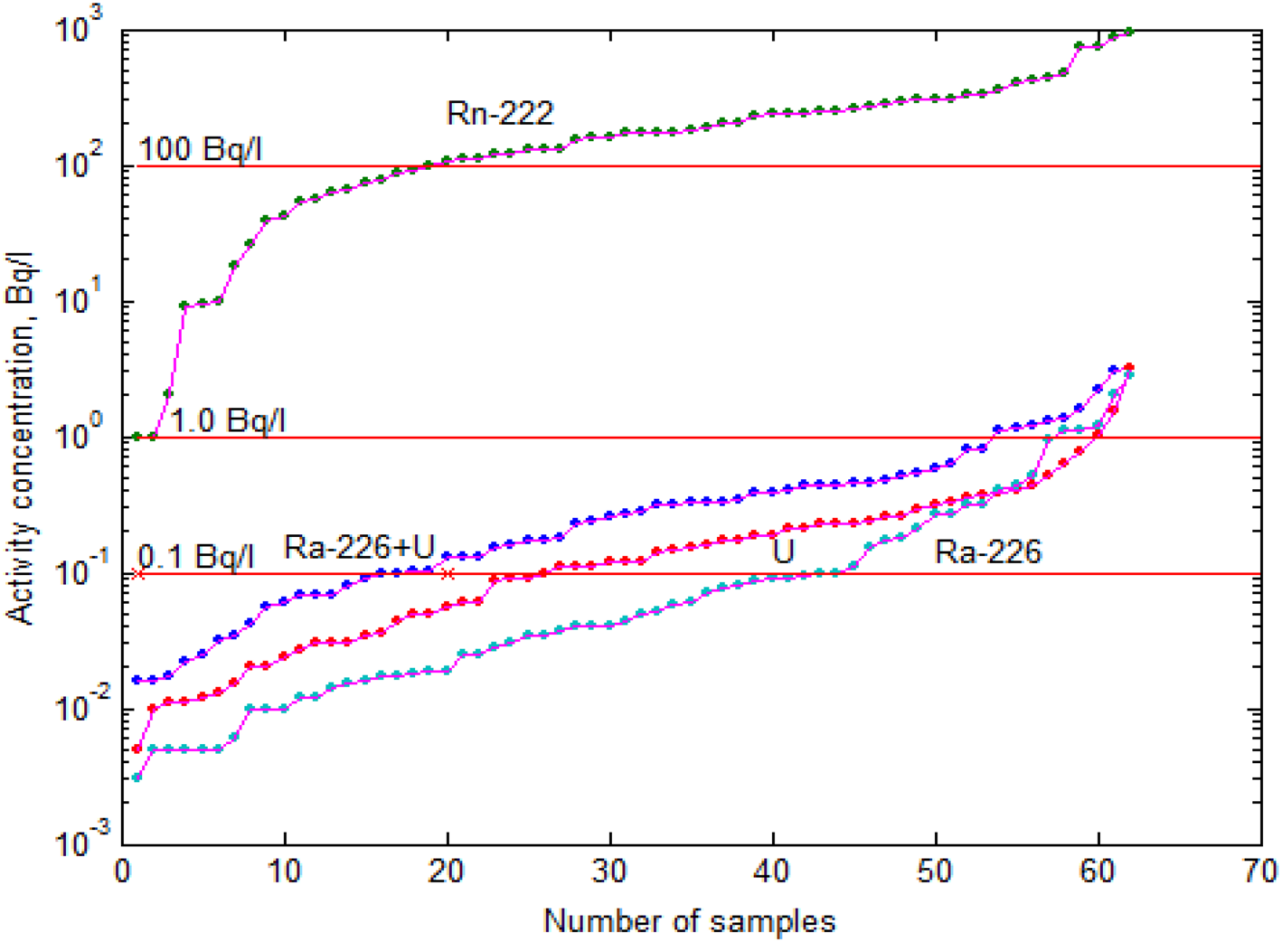
^222^Rn, ^226^Ra, ∑U, ^226^Ra + ∑U activity concentrations (each sorted separately, ascending order).

**Figure 4:**
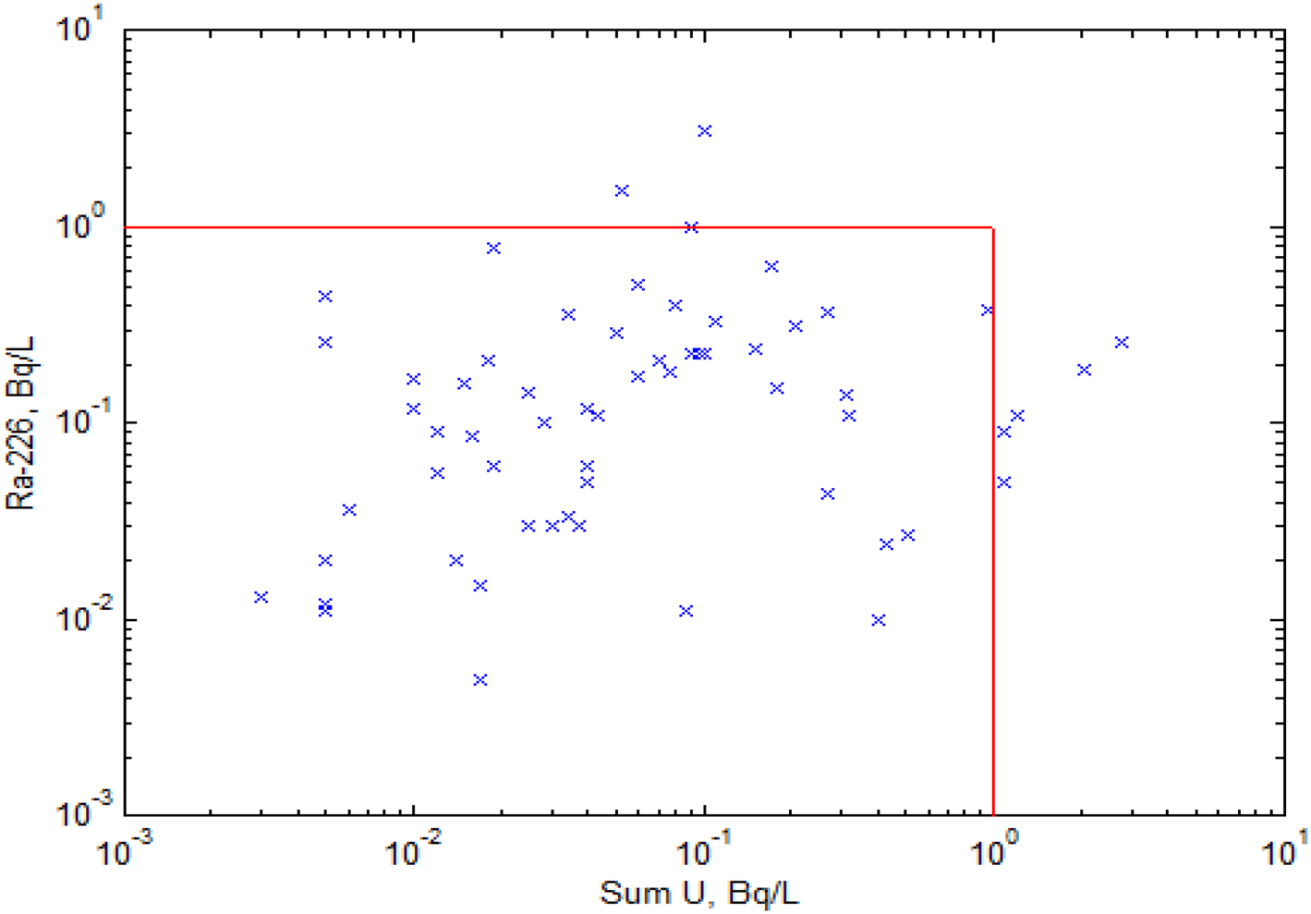
Individual sample activity concentrations pairs plot for ^226^Ra and ∑U (Bq/l). Vertical and horizontal lines set frame of 1.0 Bq/l permissible level (SanPiN)^8^.

Zhytomyr region generalized data (average, standard deviation, maximum), according to the Table 1, were among highest both for ^226^Ra and ∑U activity concentration. Earlier, we mentioned how impressive are our data presented recently^33^ obtained when implementing water purification systems (Zhytomyr and suburb). Here we consider separately an extended set of 62 samples, see Table 1, Figs. 3 and 4. It is noteworthy that the subset has average and standard deviation close to the general Zhytomyr region set for the entire observation period.

When evaluating this data subset, we sorted the obtained results of the activity concentration of ^222^Rn, ^226^Ra, ∑U, as well as the sum of ^226^Ra + ∑U (each separately, ascending order) and placed them on the Fig. 3. For ease of evaluation, we built horizontal lines corresponding to the permissible levels of (SanPiN-2010)^8^: 100 Bq/l—for ^222^Rn, 1.0 Bq/l—for ^226^Ra and for the ∑U and 0,1 Bq/l—for the calculated sum ^226^Ra + ∑U.

According to the data shown on Fig. 3 priority of this particular research is ^222^Rn, as far as the ^222^Rn activity concentration exceeds the permissible level of 100 Bq/l for the vast majority of samples (71%), when permissible level for ^226^Ra is exceeded in 3 cases (< 5%), and for ∑U—in 6 cases (< 10%). At the same time, Fig. 3 show that sum ^226^Ra + ∑U in only 15 cases (< 25%) is below the level of 0.1 Bq/l, which indicates that it is impractical to investigate the total alpha activity for such a set of research.

Figure 4 shows ^226^Ra and ∑U activity pairs for each of the 62 samples, with the horizontal and vertical lines limiting the permissible level of 1 Bq/l. Individual pairs of ^226^Ra and ∑U activity show no correlation between parent and daughter radionuclides, similar to mentioned above^2^.

### Dose estimation for water samples

**Figure 5:**
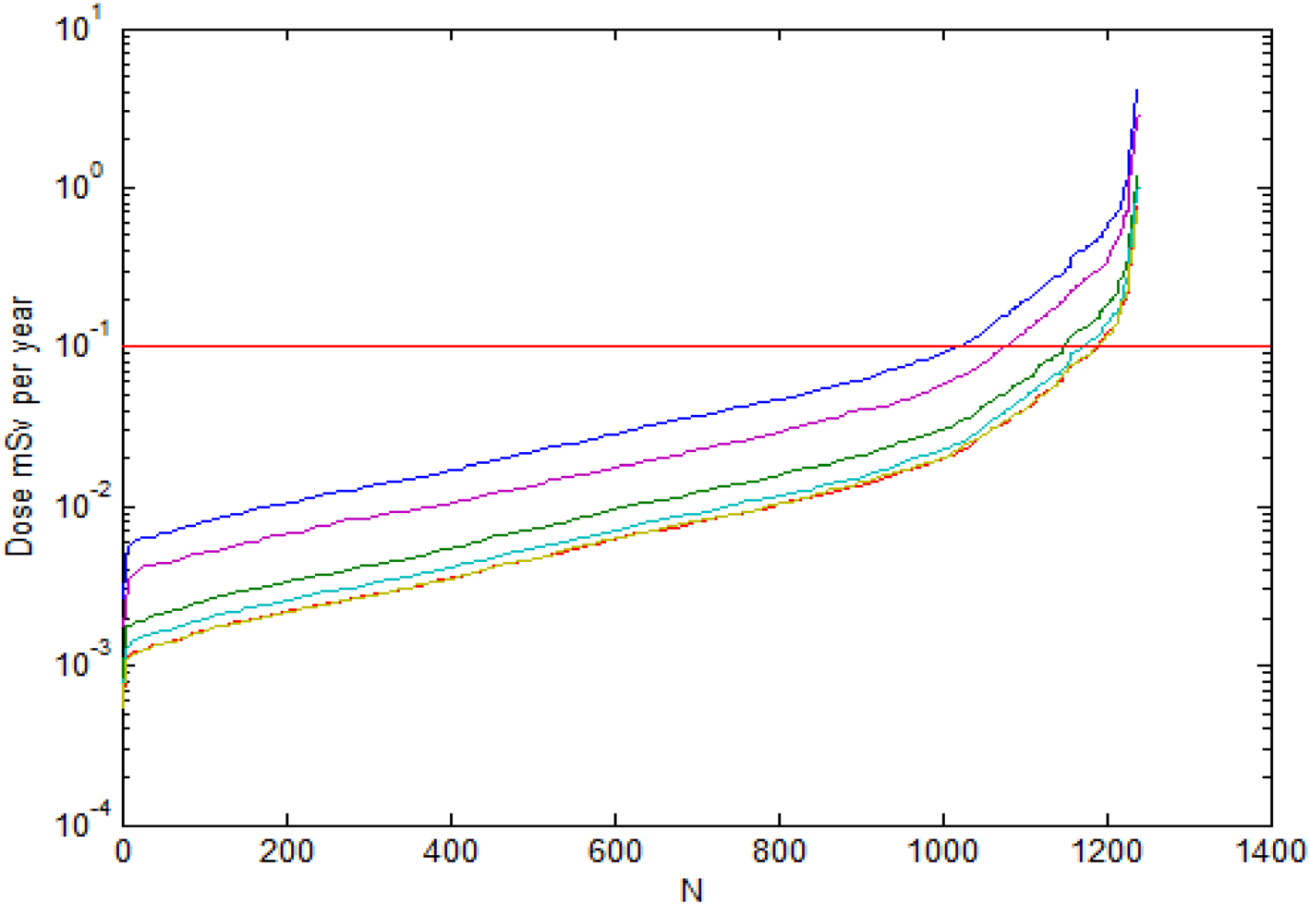
Distribution (ascending order) of irradiation doses, which were calculated for individual water samples and for representatives of different age groups. Curves correspond to age groups, from top to bottom: children under 1 year, adolescents 12–17 years, children aged 1–2 years, children aged 7–12 years, adults and children 2–7 years old.

We consider hypothetical exposure dose and for this, each sample corresponds to separate water source. For the dose assessment we use age-dependent dose coefficients for ∑U and for ^226^Ra for six age groups of the population (Council Directive 96/29/EURATOM)^25^, see Table 2. Table 3 shows generalized dose data for each age group, when at a Fig. 5 are shown dose distributions (ascending order). Each curve correspond to one age group, from top to bottom: children under 1 year, adolescents 12–17 years old, children aged 1–2 years, children aged 7–12 years, adults. The last two practically coincide visually.

**Table 2:** Dose factors (Sv/Bq) (96/29/EURATOM)^25^.

Age group	Annual water consumption, L	^226^Ra	^238^U	^234^U	Weighting factor
Children < 1 year	250	4.70E − 06	3.40E − 07	3.70E − 07	0.014
1–2 year	350	9.60E − 07	1.20E − 07	1.30E − 07	0.014
2–7 year	350	6.20E − 07	8.00E − 08	8.80E − 08	0.071
7–12 year	350	8.00E − 07	6.80E − 08	7.40E − 08	0.071
12–17 year	540	1.50E − 06	6.70E − 08	7.40E − 08	0.071
Adults	730	2.80E − 07	4.50E − 08	4.90E − 08	0.757

**Table 3:** Pattern of individual annual irradiation doses (mSv) caused by water consumption calculated for different age groups of the population^25^, (1240 samples).

Age group	Avg	SD	MinD	MaxD	Excess	Percent (%)
Children under 1 year	0.11	0.33	0.0027	4.29	220	17.7
Children 1–2 years old	0.034	0.097	0.0008	1.23	92	7.4
Children 2–7 years old	0.022	0.063	0.0005	0.79	48	3.9
Children 7–12 years old	0.026	0.079	0.0007	1.02	70	5.6
Adolescents 12–17 years old	0.07	0.23	0.0018	2.95	163	13.1
Adults	0.022	0.061	0.0005	0.75	51	4.1
Lifetime average annual dose	0.028	0.079	0.007	0.99	76	6.13

According to Table 3 dose excess of WHO recommended level of 0.1 mSv per year is varying from 3.9% to 17.7% of total sample set depending on age group.

It should be mentioned that this data set includes 150 tests that were carried out for industrial customers to check compliance water radioactivity with the requirements of the Council Directive 98/83/EC. Here some other radionuclides were also investigated. For this we calculated corresponding indicative dose for adults, it showed no any excess of the WHO recommended 0.1 mSv per year.

### Data comparison

At first we compare our current ^226^Ra and ∑U data with a previous study^1^ held in early 1990st—now 1240 compared to 520 samples then. Total sets data are comparable by maxima and maxima for some of the regions belonging to Ukrainian shield: Kyiv, Ghytomyr, Vinnytsia^27^. Different minima caused by presence in current set purified water, different (wider) geography of sampling and application of more sensitive and precise methods in our current study.

When comparing our data to data of other locations, we consider at first national wide studies. Thus, Sweden according^39^, have many geological oddities including environmental radioactivity, and there radioactivity accompanying drinking water as well. Water radioactivity is relatively low in dug wells, when drilled wells give moderate activities of ^222^Rn—203 ± 86 Bq/l, ^226^Ra—0.09 ± 0.01 Bq/l and Uranium—14.3 ± 4 μg/L as average in normal Precambrian rocks. Only uranium-rich granites and pegmatites give high ^222^Rn—300–4000 or 18,000–55,000 Bq/l, ^226^Ra—0.05–0.8 or 0.35–2.5 Bq/l and U—up to 268 μg/L.

Similarly in Finland according to Asikainen^40^ average water radioactivity at waterworks is relatively low—25 Bq/l of ^222^Rn and 0.004 Bq/l of ^226^Ra in average. Higher radioactivity of drilled wells—630 Bq/l of ^222^Rn and 0.11 Bq/l of ^226^Ra in average, when considering drilled wells they may contain abnormally high U up to 21 mg/l or even higher. Later Vesterbacka^41^ estimate the mean annual effective dose from natural radionuclides for users of drilled wells to be 0.41 mSv, for users of wells dug in the ground 0.05 mSv and for people using water from waterworks 0.02 mSv. Main role as dose forming factor in wells water play ^222^Rn forming up to 60–75% of irradiation. Most of dose by long-lived radionuclides cause ^210^Pb and ^210^Po, when ^226^Ra and ^228^Ra role is minor.

In Estonia^42^ activity concentration of ^226^Ra and ^228^Ra considered as highest contributors to the total indicative dose (TID) for waters of Cambrian-Vendian (Cm-V) aquifer. In study report^43^, it was concluded, that about 91% of Cm-V aquifer consumers (20% of the Estonian population) obtain high doses, i.e. TID exceeding 0.1 mSv/y.

The activity concentrations of ^238^U, ^234^U and ^210^Po in drinking water from certain sources in South-Central Bulgaria reported recently by Ref.^44^. The results obtained varied from 79 to 826 mBq/l for ^238^U, 130 to 1623 mBq/l for ^234^U, < 0.5 to 25.5 mBq/l for ^210^Po. Corresponding annual effective dose varied from 8.9 to 62.5 μSv/y with a mean of 30.1 μSv/y. It is below the individual dose criterion of 100 μSv/y. The highest contribution to the annual effective dose was found to be ^234^U.

In Ireland during of 2007–2011 243 public water supply sources was tested^45^ and in all total beta fit requirement 1.0 Bq/l, actual max 0.55 Bq/l results of total alpha in 175 cases (86%) comply with limit 0,1 Bq/l. Among 14% samples highest of alpha is 0.24 Bq/l. ^222^Rn in all samples is below 500 Bq/l—national recommended level, while highest is 345 Bq/l. no correlation observed between uranium or ^222^Rn with alpha activity of samples exceeding 0.1 Bq/l.

In Poland drinking water radioactivity and corresponding irradiation is warring wide from low for Warsaw^46^, where considered ^210^Po, ^234^U and ^238^U isotopes as for surface 0.12, 3.91 and 2.75 mBq/l and for deep-water intakes were 0.25, 0.24 and 0.20 mBq/l, respectively. The annual dose absorbed because of the consumption of drinking water by an inhabitant is below 0.5 μSv/y. Studies of natural radioactivity in spring water in the Sudety Mountains^47^, where uranium exploration was conducted in the early 1950s. Annual effective doses due to radionuclide intake calculated for 4 out of 20 spring waters used for consumption by spa patients and inhabitants without ^222^Rn were of the range 0.4 μSv to 9.2 μSv, for patient for of a 20-day duration stay and from 1.3 μSv/y to 26.7 μSv/y for an inhabitant. The contribution of radon consumed with water raises these values to 209.4 μSv per 20 days and 608.3 μSv/y for a patient and inhabitant, respectively. This shows how water ingestion dose may highly varying in certain places depending on considered factors of irradiation and for different categories of public.

Geological conditions bring certain composition of natural radionuclides into the groundwater, which cause corresponding internal human exposure when opportunities for consumption of this water created.

## Conclusions

The aim of our work was generalizing the activity concentration of ^226^Ra and the ∑U in a set of 1240 drinking water samples tested in the laboratory for the period 1998–2023.

Comparison of the data set with previous studies^2^ shows that the sample set is wider in terms of sampling locations; the averages here are lower, although the sample resembles the previous one in terms of regional maxima.

The consolidated table clearly shows the regions where the concentration of activity is higher, in particular, where the regulations exceeded.

The given data show the expediency of researching water for radioactivity when assessing the need for water purification in high radiation areas, in particular, for Zhytomyr, where excess of permissible level was 71% for ^222^Rn, 5%—for ^226^Ra, and 10%—for ∑U.

The assessment of the average annual irradiation dose from lifetime water consumption performed for the sample set shows that the WHO recommended dose limit (0.1 mSv per year) exceeded here for 76 cases or 6.13% (Supplementary Information).

### Supplementary Information

Supplementary Information.
